# Associations Between Objective Television Exposure and Cancer Perceptions in a National Sample of Adults

**DOI:** 10.1177/1073274819846603

**Published:** 2019-05-26

**Authors:** Caitlin G. Allen, Colleen M. McBride, Regine Haardörfer, Megan C. Roberts

**Affiliations:** 1 Behavioral Sciences and Health Education Department, Rollins School of Public Health, Emory University, Atlanta, GA, USA; 2 Division of Cancer Control and Population Sciences, The National Cancer Institute, Bethesda, MD, USA

**Keywords:** cancer perceptions, cancer prevention, cancer, media, health education

## Abstract

The expanding sources of media coverage of cancer may have a powerful impact on emotions, cancer knowledge, information seeking, and other health behaviors. We explored whether television advertisements were associated with cancer worry, perceived risk, and perceived ability to prevent cancer using cross-sectional data from the Health Information National Trends Survey (HINTS) linked to television advertisement data from Kantar Media. We conducted hierarchical linear modeling assessing 2-level models for each of the 3 outcomes of interest. The most common content included advertisements for cancer clinics (54.4%), public service announcements about cancer (22.0%), and advertisements about cancer organizations (9.1%). Most variance in cancer perceptions was due to individual-level characteristics and not exposure to television advertisements, which aligns with previous literature suggesting a small, but significant, association of television exposure with health beliefs. Higher levels of exposures to cancer-specific television advertisements were associated with higher levels of risk perceptions. Additionally, older adults’ levels of perceived worry and risk were more likely to be associated with television exposure than younger adults. Given the substantial investments being made in cancer advertisements on television, the differences in exposure are important to consider in future efforts to understand predictors of beliefs about cancer and in the development of interventions designed to target risk-reducing behaviors.

## Introduction

Media continues to be a mainstay strategy for disseminating health information as it enables reach to large populations.^1-5^ Studies about the influence of media on health beliefs and behaviors are often tied to the effects of notable celebrity announcements and public health campaigns on attitudes and behaviors.^6-9^ For example, public surveys following high-profile celebrity cancer disclosures have shown increased perceptions of risk and uptake of cancer screening behaviors (eg, the Angelina Jolie effect).^6,7^ However, less attention has been given to the impact of routine exposure to health information.^9-11^ This is a notable gap, as previous content analyses show that cancer-specific messages are common and growing in everyday media, particularly in television.^12^ For example, in 2014 over US$174 million was spent on cancer clinic advertisements alone, a 3-fold increase since 2005.^13^

Cultivation theory suggests that habitual exposure to messages via television shapes viewers’ conceptions of social reality.^14-16^ According to cultivation theory, there is a significant positive association between amount of exposure and message influence (ie, more exposure leads to more influence).^17^ Message exposure is thought to influence individual’s perceptions of the problem and attitude formation about actions to take. Accordingly, this theory suggests that regular incidental exposures to cancer messages, both positive and negative, may influence perceived worry, risk, and ability to prevent cancer.^18-21^ Indeed these routine exposures may elevate levels of awareness and value expectancies for adopting preventive actions. For example, there is evidence suggesting that the use of media increases the likelihood of participating in routine cancer screening such as mammograms.^22,23^ Conversely, messages in the media may prompt negative emotions including worry and fear and in turn, information avoidance (eg, decision not to seek screening).^24-29^

To date, literature exploring the association of media exposure with cancer perceptions has been based predominately on self-reported television viewing (eg, hours watching television).^11,12,16,30^ Yet, cultivation theory suggests that both subjective (eg, self-report) and objective (eg, constant exposure) indicators of television exposure are germane. Although awareness of cancer-related television messages may not be present in working memory they could still meaningfully influence cancer perceptions.

The few studies assessing *objective* exposures have primarily focused on the content of advertisements. Advertisements, defined as commercial breaks that are designed to convey a message, market a product, or service, can include a wide range of information such as public service announcements about health screening, information about specific clinics or services, and local or national events. Content analyses of local and national television cancer coverage have suggested that messages may unintentionally reinforce fatalistic beliefs about cancer, as individuals tend to choose media content that is congruent or reinforcing of their existing beliefs, attitudes, or behaviors.^31^ However, the content of these advertisements has not been directly linked to viewers’ cancer perceptions.^13,32-36^

Exposure to television advertisements also varies substantially based on sociodemographics, which could moderate the association between exposure to cancer advertisements on television and cancer perceptions.^12,37,38^ Women tend to watch more television per day (4 hours and 11 minutes) than men (average of 3 hours and 34 minutes) and older Americans (65+) watch significantly more television than their younger counterparts.^37-40^ Across racial and ethnic groups, African Americans make up the largest segment of the traditional television audience (25%), watching over 200 hours of television per month.^38,39^ Indeed, television is commonly named as a key source of information regarding medical advice, health, cancer, particularly for older adults, those without access to health care, and those with low income.^5,12,41-43^

**Figure 1.**
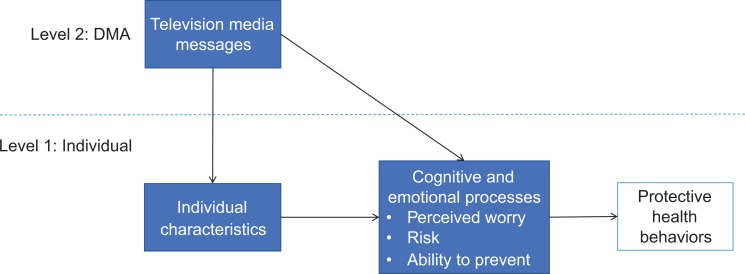
Impact of Individual- and DMA-Level Variables on Cognitive and Emotional Processes Leading to Protective Health Behaviors. This conceptual model describes the 3 research questions and potential relationships between individual- and DMA-level variables that influence cognitive and emotional processes. The cognitive and emotional processes (worry, risk, prevention) are likely to influence protective health behaviors; however, we did not test the association between cognitive and emotional processes and protective health behaviors in this article. Research questions are as follows: 1) Are individual's reported levels of cancer worry, risk, and perceived ability to prevent cancer associated with exposure to cancer-specific television advertisements?, 2) What are the individual-level and DMA-level factors associated with cancer worry, risk, and perceived ability to prevent cancer?, and 3) Do DMA-level factors (exposure to cancer television advertisements and dollars spent on these advertisements) moderate the association of individual-level demographic variables (age, race, gender) with cancer worry, risk, and perceived ability to prevent cancer?

The substantial investment made in cancer advertising together with American’s ubiquitous television viewing raises questions about the extent to which routine exposure to cancer advertisements influences cancer perceptions known to be associated with risk reducing behaviors. In this report, we consider whether, and through what mechanisms, objective television exposure may be associated with individual viewers’ levels of worry, risk, and perceived ability to prevent cancer (Figure 1). Television media exposure is assessed based on designated marketing areas (DMAs) that represent catchment areas where people are exposed to similar cancer-related television marketing content. Previous literature has suggested that variation in broadcasting, measured through DMA programming is important when assessing cancer-related risk perceptions and, in turn, cancer prevention behaviors.^11,12^ The DMA-level assessment enables differences in cancer-related television exposure between and within market contexts to be considered.^11,12^

Specifically, we sought to answer the following questions about the role of cancer-specific television exposure on 3 outcomes of interest (worry, risk, and perceived ability to prevent cancer): 1) Are individual's reported levels of cancer worry, risk, and perceived ability to prevent cancer associated with exposure to cancer-specific television advertisements?, 2) What are the individual-level and DMA-level factors associated with cancer worry, risk, and perceived ability to prevent cancer?, and 3) Do DMA-level factors (exposure to cancer television advertisements and dollars spent on these advertisements) moderate the association of individual-level demographic variables (age, race, gender) with cancer worry, risk, and perceived ability to prevent cancer?

## Materials and Methods

### Analytical Sample

We used cross-sectional data from the HINTS 4 cycle 3, a population-based survey administered by the National Cancer Institute.^44^ This nationally representative survey is designed to better understand the public’s need for access to and use of cancer-related health information. Data from HINTS 4 cycle 3 were combined with DMA data available from Kantar Media (2013), which provides detailed information about marketing strategies based on DMAs.^45^ Data sources were linked by common DMA. Individuals were included in the analytic sample if they responded to one of the outcomes of interest from HINTS (perceived cancer worry, risk, and perceived prevention).

### Variables

#### Individual-level outcome variables

The 3 outcomes of interest were *cancer worry*, assessed on a 5-point Likert scale by the question, “How worried are you about getting cancer in your lifetime” (not at all, slightly, somewhat, moderately, extremely), *perceived risk* of getting cancer, assessed on a 5-point Likert scale with the question, “Compared to other people your age, how likely are you to get cancer in your lifetime?” (very unlikely, unlikely, neither likely or unlikely, likely, very likely), and beliefs about *possibility of prevention*, assessed on a 4-point scale by the question, “How much do you agree or disagree: There is not much you can do to lower your chances of getting cancer?” (strongly agree, agree, disagree, strongly disagree). Only individuals who answered “no” to the question, “Have you ever had cancer” were asked questions about cancer worry, risk, and perceived prevention.

#### Individual-level independent variables

Demographic variables included in this study were age, race, income, level of education, and gender. Race and ethnicity were dichotomized as non-Hispanic white or other races (ie, Hispanic, non-Hispanic black, non-Hispanic American Indian or Alaska Native, non-Hispanic Asian, non-Hispanic Native Hawaiian, and non-Hispanic multiple races). Household income was categorized as less than US$35 000, between US$35 000 to US$74 999, and US$75 000 or more. Education level was assessed using the following categories: less than high school, high school graduate, some college, bachelor’s degree, and postbaccalaureate degree. Health Information National Trends Survey documentation indicates multiple imputation was completed for all missing demographic variables.^44^

In addition to demographic variables, we included individual reported trust in sources of information.^46^ Trust in health-related information delivered on local television was assessed with the question, “In general, how much would you trust information about health or medical topics from local television” (a lot, some, a little, not at all); trust in national television was assessed with the question, “In general, how much would you trust information about health or medical topics from national or cable television news programs” (a lot, some, a little, not a lot). These were combined and categorized as high (a lot), medium (some or a little), or low (not at all) television trust.

#### Designated Market Area-level independent variables

The full sample of DMAs was included in the HINTS data set (N = 210). The DMAs represent television media markets where people receive the same or similar television and radio offerings and content. These often overlap with large cities but can also cut across multiple metropolitan areas, especially in rural regions. The DMAs are used to help broadcasters plan and determine strategies for advertisement and campaign performance. Thus, we measured cancer-specific media across DMAs using data available from Kantar Media.^45^ Total exposure to cancer-related advertisements was measured in seconds per year and included information about advertisements that were specific to local DMAs and national coverage. The total seconds of DMA exposure at the local and national levels were used for this analysis. Similarly, total dollars spent on cancer-related advertisements were measured based on dollars spent in thousands per year. Both seconds and dollars spent at the DMA and national levels were used for this analysis. Specific cancer advertisement types included clinical, public service announcement, other, events, cancer organization, type of cancer, vignette, and foundation, as designated by Kantar Media.^45^

### Statistical Analysis

Data were analyzed with SAS version 9.4. To account for probability sampling design and jackknife replicate weights, univariate frequencies and means were weighted to obtain nationally representative estimates for descriptive statistics. Bivariate analyses were assessed for all outcomes and predictors, which accounted for DMA-level clustering (results are reported in Supplemental Table 1).

Our final analysis tested multiple outcomes with 3 different models (perceived worry, comparative risk, and ability to prevent cancer). These tests were conducted without population weights to maximize sensitivity and power.^11^ We conducted hierarchical linear modeling assessing 2-level models for 3 different outcomes of interest: perceived risk, worry, and ability to prevent cancer. Continuous predictor variables (age, exposure to cancer advertisements, dollars spent on cancer advertisements) were grand mean centered. All individual- and DMA-level predictors were included in the final model, and a compound symmetric correlation/covariance matrix was specified. We used a sequential modeling strategy with fixed slopes and random intercepts. We estimated (1) an unconditional model, (2) a model with individual-level predictors, and (3) a model with individual- and DMA-level factors (presented in Supplemental Table 2), and (4) a model assessing selected cross-level interaction effects. The final model for each outcome included all 3 interaction terms (exposure to cancer advertisements by age, gender, race) and is presented in the results. We assessed model fit using QIC. We used complete case analysis where individuals who responded to the outcome of interest were included. We also assessed for multicollinearity of individual-level predictors using the variance inflation factor.

## Results

There were a total of 2565.46 hours of local and national cancer advertisements of the year equivalent to 307,855.2 thirty-second advertisements. The most common type of cancer advertisements related to services of cancer clinics (54.4%), public service announcements about cancer (22.0%), and cancer organizations (9.1%; Table 1).

**Table 1.** Total Hours of Cancer Advertisements by Type.

Type of Cancer Advertisement	Total Hours
	N (%)
Prostate	2.28 (0.09)
Vignette	31.56 (1.25)
Lung	40.88 (1.62)
Foundation	49.20 (1.95)
Event	61.87 (2.45)
Other	179.37 (7.10)
Cancer organization	229.93 (9.10)
Public service announcement	556.75 (22.04)
Clinic	1374.62 (54.41)
Total	2526.46 (100.00)

The HINTS sample was largely non-Hispanic white (66.9%) and college educated (65.9%); 52% of the sample was female with an average age 45.3 (standard deviation [SD] = 0.2). Three-quarters of individuals had “moderate” trust in health messages on television (77.6%; Supplemental Table 1). The average level of perceived cancer worry (5-point Likert scale, “How worried are you about getting cancer in your lifetime?”) was 2.4 (SD = 0.04, scale 1-5). Overall, individuals perceived themselves to be at average risk for cancer (2.8, SD = 0.03, scale 1-5; “Compared to other people your age, how likely are you to get cancer?”). Participants tended to disagree with the statement, “There’s not much you can do to lower your chances of getting cancer” (2.9, SD = 0.03, scale 1-4).

### Amount of Worry, Risk, and Perceived Ability to Prevent Cancer Attributed to Television Exposure

Unconditional hierarchical linear models indicated that DMA-level exposure (seconds or dollars) to cancer-specific advertisements accounted for very little variance in cancer worry (1%), comparative risk perceptions (1.2%), or perceived prevention (<1%).

### Individual- and DMA-Level Factors Associated With Cancer Perceptions

All bivariate associations are included in Supplemental Table 1, and the direct effects hierarchical model for each outcome is included in Supplemental Table 2. The final model that includes relevant interaction effects is presented in Table 2.

**Table 2.** Final Hierarchal Linear Models for Individual’s Perceived Worry, Comparative Risk, and Ability to Prevent Cancer.^a^

	Perceived Worry			Comparative Risk			Ability to Prevent		
	β	SE	*P* Value	β	SE	*P* Value	β	SE	*P* Value
Intercept	2.197	0.105	<.0001	2.682	0.122	<.0001	2.776	0.099	<.0001
Designated marketing area									
Exposure to cancer ads (hours)	0.022	0.050	.654	**0.079**	**0.038**	**.038**	0.021	0.003	.524
Dollars spent on cancer ads (million)	−0.367	0.933	.694	−0.376	0.226	.096	−0.021	0.028	.452
Individual characteristics									
Race/ethnicity									
Non-Hispanic white	0.094	0.059	.110	**0.254**	**0.047**	**<.0001**	**0.170**	**0.040**	**<.0001**
Other (ref)									
Gender									
Male (ref)									
Female	**0.155**	**0.042**	**.0002**	**0.018**	**0.038**	**.643**	−0.028	0.038	.567
Age	**−0.009**	**0.002**	**<.0001**	**−0.008**	**0.083**	**<.0001**	**0.003**	**0.001**	**.039**
Television trust									
Low (ref)									
Medium	0.042	0.094	.656	0.057	0.083	.491	−0.143	0.083	.084
High	−0.135	0.125	.281	0.171	0.099	.087	−0.170	0.110	.121
Interactions									
Age × exposure (hours)	**0.003**	**0.001**	**.018**	**0.002**	**0.001**	**.028**	0.001	0.001	.649
Gender × exposure (hours)	0.038	0.042	.367	−0.003	0.030	.938	−0.014	0.039	.716
Race × exposure (hours)	−0.033	0.048	.488	−0.025	0.043	.557	−0.021	0.028	.452
Model fit									
QIC	1989.388			1959.999			2299.137		

*Note*: Bold values indicate *p* < 0.05.

^a^ Models adjusted for household income and education level.

#### Perceived worry

There were no significant associations between DMA media exposure (seconds or dollars) and cancer worry. When accounting for DMA-level variance, there were significant differences in perceived worry by gender and age (Table 2). Women were more likely than men to be worried about cancer (β = 0.155; *P* = .0002). Level of worry also decreased as individuals aged (β = −0.009; *P* < .0001).

#### Comparative risk

When accounting for DMA-level variance, there were differences in comparative risk perceptions by race/ethnicity, with non-Hispanic whites being more likely to feel at risk that other racial groups (β = 0.254; *P* < .0001). Older individuals were less likely to feel at risk than younger individuals (β = −0.008; *P* < .0001). As exposure to cancer advertisements increased, individuals were more likely to believe they would get cancer (β = 0.079; *P* = .038; Table 2).

#### Ability to prevent cancer

There were no significant associations between DMA-level exposure and perceived ability to prevent cancer. At the individual-level, white participants believed more strongly in their ability to prevent cancer compared to nonwhite individuals (β = 0.170; *P* = <0.0001). Older adults believed more strongly in their ability to prevent cancer than younger individuals (β = 0.003; *P* = .039; Table 2).

### Moderators of Individual Cancer Perceptions

**Figure 2.**
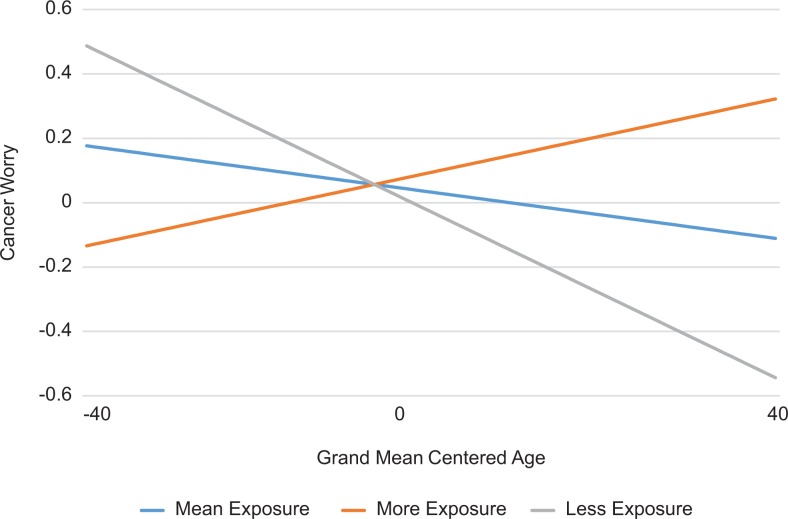
Interaction effect of age and television exposure on cancer worry. The differences in frequency of cancer worry based on mean and standard deviations of exposure across designated marketing area (DMA).

**Figure 3.**
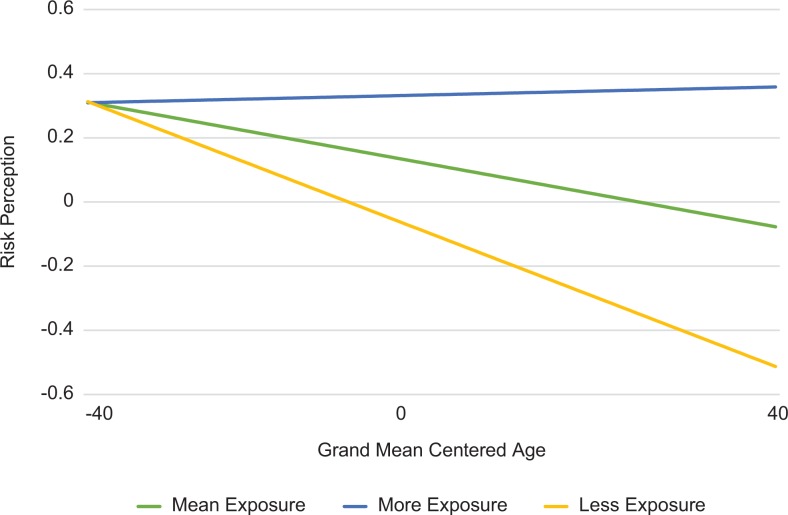
Interaction effect of age and television exposure on comparative risk perceptions. The differences in frequency of risk perceptions based on mean and standard deviations of exposure across designated marketing area (DMA).

There were no interaction effects between gender and exposure to cancer advertisements or race and exposure to cancer advertisements on any outcomes. However, there was a significant interaction between age and amount of exposure on worry (β = 0.003; *P* = 0.018; Table 2, Figure 2) and comparative risk (β = 0.002; *P* = .028; Table 2, Figure 3). The association of reported cancer worry with DMA-level exposure indicated that older viewers’ cancer worry was more likely to be associated with cancer media exposure than younger individuals’ worry. This association was consistent in comparative risk; older viewers’ comparative risk perceptions were more likely to be associated with cancer media exposure than younger individuals’ perceived risk.

## Discussion

Our aims in this study were to understand whether cancer-specific television exposures (measured through objective broadcast seconds and dollars spent on cancer advertisements across DMAs) were associated with individual’s level of worry about cancer, perceived risk of developing cancer, and perceived control over developing cancer.

### Cancer Perceptions Largely Were Not Associated With Exposure to Television Advertisements

We found that exposure to cancer television advertisements explained little of the variance (<2%) in individual-level worry about cancer, perceived risk of developing cancer, or perceived ability to prevent cancer. These analyses are among the first to date that have considered the association between objective exposure to cancer advertisements and subjective outcomes related to cancer beliefs. Literature has predominantly discussed the role of specific media campaigns designed for certain interventions on behavior changes, finding that targeted campaigns can positively influence health behaviors (or prevent negative changes in health behaviors) related to cancer.^8,9^ Although some of the advertisements included in this report may have been part of specific health campaigns, our primary focus was on routine exposure to cancer messaging.

It is notable that when controlling for sociodemographic variables, exposure to cancer advertisements was only significantly associated with risk perceptions (not perceived worry or ability to prevent cancer). Although overarching patterns of television content may support beliefs, specific genres of content may be more or less consequential than others. For example, previous studies focused on cancer advertising content found that much of the media coverage focused on fear-based emotional appeals and cancer treatments, rather than prevention.^13^ Prior studies also have had mixed evidence related to whether risk perceptions are associated with behavior changes. Most conceptual models suggest that perceiving oneself to be at risk can be a cue to behavior change; however, unduly heightened risk perceptions can also cue motivated processing of information and prompt distancing and denial of risk. Thus, it will be important for future research to include more information about the content and costs of advertisements as they relate to cancer perceptions.^47-49^

Time exposed to cancer advertisements was not associated with worry or perceived ability to prevent cancer. Although generally correlated, worry, risk, and perceived ability to prevent cancer are considered distinct and independent predictors of behaviors.^50^ Past studies found that most advertisements place high literacy demands on viewers (10th grade) and that content is often not perceived to be aligned with average people.^34^ Thus, the lack of association of advertisements with worry and perceptions about prevention could be due to individual’s poor understanding of the advertisements or unrelatable content. These are important consider prevention, as the construct of perceived control (ie, ability to take action to prevent cancer) is associated with predictions of behaviors and identifying motivations.^51^

### Designated Marketing Area-Level Factors Moderate the Association of Age With Cancer Perceptions

Exposure to television was associated with cancer worry only for older adults; likewise perceptions of comparative risk were higher for older than younger adults with the same amount of exposure to television. This is particularly interesting, as older adults reported lower levels of cancer worry and less perceived risk than younger adults. The direction of this association is unclear, given the cross-sectional nature of the data. It may be that older adults with heightened cancer worry or risk are more inclined to pay attention to cancer advertisements than those who worry less about cancer or perceive themselves to be at risk. Alternatively, greater exposure to cancer advertisements could have increased worry and risk perceptions among older adults but not younger adults.

The majority (54.4%) of cancer advertisements reported in the DMA data were for cancer treatment centers. Previous studies have found the content of cancer treatment advertisements draws heavily on emotional appeals (ie, hope and fear) in order to promote uptake of their services, with little substantive information.^52^ Such messages may be more salient for older adults. Future observational studies could employ quasi-experimental design methods to better assess the direction of the relationship between the exposure and various cancer-beliefs and cancer behavior outcomes. For example, using propensity score matching techniques could help match DMAs based on key characteristics and assess for variation in beliefs based only on differences in exposure to television advertisements while controlling for all other observed similarities.

The absence of association of exposure to television advertisements among younger adults where the potential for cancer prevention is greatest suggests that cancer messaging may be missing its mark among important demographic groups. The content and mode of delivering (eg, television vs social media) these messages may not be appealing to younger audiences as they tended to focus on treatments of cancers and promotion of specific cancer centers.^13,34^ Trends also suggest that the majority of the investment in cancer advertisements is still occurring through television media despite growing use of social media and streaming among younger generations.^13^ Given that we found little association of these advertisements with young individuals’ cancer-related perceptions, substantial resources may be being invested with little to no benefit for prevention among those who could benefit most. In fact, previous studies have suggested that there are considerable discussion of cancer occurring in other spaces such as Twitter and Facebook.^53-56^ Thus, a better investment for reaching younger generations with prevention-related messages may be found in alternative channels.

### Limitations

Findings from this study are not without limitations. First, this study focused on exposure to television cancer advertisements (not comprehension of messages or quality of messages). This is an important limitation because we were unable to identify nuances in whether the type of advertisement was associated with each outcome of interest.^57^ Second, we focused only on television as the media source. Although television continues to be the most common form of media exposure for Americans, there is growing use of other information sources outside of television that could influence beliefs (eg, social media and streaming.).^38,39^ Further research is needed to better identify how each source of media is used among the public and whether there are differences in the impact of these communication channels on cancer information-seeking and health behaviors based on each of these sources.^58^ Next, we focused on identifying associations across the US population. There could be differences based on variables that were not included in our model, such as rural and urban differences, and personal cancer risk. Future studies could further assess both DMA-level factors (eg, rural and urban) and individual-level factors (eg, personal cancer history) on outcomes of interest by including them in hierarchical models or stratifying by these variables. Finally, higher level DMA variables explained a small amount of the variance in each outcome; however, this may be due to small cell size at the cluster level (DMA). Despite the limited contribution of DMA-level variables on the model, it was important to account for these differences in levels of exposure using multilevel modeling techniques.^59^

## Conclusions

This study contributes to our understanding of objectively measured television exposure to cancer advertisements and cancer perceptions. Given the considerable cost and effort of cancer advertisements, the lack of discernable associations of exposure with key cancer perceptions, particularly among young adults where cancer prevention opportunities are greatest, is a missed opportunity. Further prospective research is needed to ascertain whether cancer-related advertisement content influences cancer perceptions. Additionally, as other forms of media become more prevalent, it will be important to monitor and assess the messages being presented in those channels.

## Supplemental Material

Supplemental Material, Supplemental_Table_1_and_2 - Associations Between Objective Television Exposure and Cancer Perceptions in a National Sample of Adults Click here for additional data file. Supplemental Material, Supplemental_Table_1_and_2 for Associations Between Objective Television Exposure and Cancer Perceptions in a National Sample of Adults by Caitlin G. Allen, Colleen M. McBride, Regine Haardörfer and Megan C. Roberts in Cancer Control

## References

[1] Katapodi MC Lee KA Facione NC Dodd MJ Predictors of perceived breast cancer risk and the relation between perceived risk and breast cancer screening: a meta-analytic review. Prev Med. 2004;38(4):388–402. 15020172 10.1016/j.ypmed.2003.11.012

[2] Schroy PC III Glick JT Robinson PA Lydotes MA Evans SR Emmons KM Has the surge in media attention increased public awareness about colorectal cancer and screening? J Commun Health. 2008;33(1):1–9. 10.1007/s10900-007-9065-5 18080203

[3] Lowery JT Ahnen DJ Schroy PC III Understanding the contribution of family history to colorectal cancer risk and its clinical implications: a state-of-the-science review. Cancer. 2016;122(17):2633–2645. 27258162 10.1002/cncr.30080 PMC5575812

[4] Niederdeppe J Frosch DL Hornik RC Cancer news coverage and information seeking. J Health Commun. 2008;13(2):181–199. 18300068 10.1080/10810730701854110 PMC2970505

[5] Yanovitzky I Blitz CL Effect of media coverage and physician advice on utilization of breast cancer screening by women 40 years and older. J Health Commun. 2000;5(2):117–134. 11010345 10.1080/108107300406857

[6] Evans DG Wisely J Clancy T Longer term effects of the Angelina Jolie effect: increased risk-reducing mastectomy rates in BRCA carriers and other high-risk women. Breast Cancer Res. 2015;17:143. 26603733 10.1186/s13058-015-0650-8 PMC4659163

[7] Troiano G Nante N Cozzolino M The Angelina Jolie effect—Impact on breast and ovarian cancer prevention. A systematic review of effects after the public announcement in May 2013. Health Educ J. 2017;76(6):707–715.

[8] Wakefield MA Loken B Hornik RC Use of mass media campaigns to change health behaviour. Lancet. 2010;376(9748):1261–1271. 20933263 10.1016/S0140-6736(10)60809-4 PMC4248563

[9] Brown JD Walsh-Childers K Effects of media on personal and public health In: Bryant J Zillman D, eds. Media Effects: Advances in Theory and Research. Hillsdale, NJ: Lawrence Erlbaum Associates; 1994:389–415.

[10] Stryker JE Moriarty CM Jensen JD Effects of newspaper coverage on public knowledge about modifiable cancer risks. Health Commun. 2008;23(4):380–390. 18702002 10.1080/10410230802229894

[11] Lee CJ Niederdeppe J Genre-specific cultivation effects: lagged associations between overall TV viewing, local TV News viewing, and fatalistic beliefs about cancer prevention. Communic Res. 2011;38(6):731–753. 25605981 10.1177/0093650210384990 PMC4297652

[12] Niederdeppe J Fowler EF Goldstein K Pribble J Does local television news coverage cultivate fatalistic beliefs about cancer prevention? J Commun. 2010;60(2):230–253. 20563221 10.1111/j.1460-2466.2009.01474.x PMC2885705

[13] Vater LB Donohue JM Park SY Schenker Y Trends in cancer-center spending on advertising in the United States, 2005 to 2014. JAMA Intern Med. 2016;176(8):1214–1216. 27400275 10.1001/jamainternmed.2016.0780 PMC4969207

[14] Slater D Elliott WR Television’s influence on social reality. Q J Speech. 1982;68(1):69–79.

[15] Gerbner G Cultivation analysis: an overview. Mass Commun Soc. 1998;1(3-4):175–194.

[16] Shim M Kelly B Hornik R Cancer information scanning and seeking behavior is associated with knowledge, lifestyle choices, and screening. J Health Commun. 2006;11(suppl 1):157–172. 16641081 10.1080/10810730600637475

[17] Morgan M Shanahan J Two decades of cultivation research: an appraisal and meta-analysis. Commun Yearb. 1997;20(1):1–45.

[18] Shapiro S The effects of incidental ad exposure on the formation of consideration sets. J Consum Res. 1997;24(1):94–105.

[19] Shapiro S When an ad’s influence is beyond our conscious control: perceptual and conceptual fluency effects caused by incidental ad exposure. J Consum Res. 1999;26(1):16–36.

[20] Carlsson ME Cancer patients seeking information from sources outside the health care system: change over a decade. Eur J Oncol Nurs. 2009;13(4):304–305. 19362053 10.1016/j.ejon.2009.03.005

[21] Dutta-Bergman MJ Primary sources of health information: comparisons in the domain of health attitudes, health cognitions, and health behaviors. Health Commun. 2004;16(3):273–288. 15265751 10.1207/S15327027HC1603_1

[22] Wallington SF Oppong B Iddirisu M Adams-Campbell LL Developing a mass media campaign to promote mammography awareness in African American women in the nation’s capital. J Community Health. 2018;43(4):633–638. 29280089 10.1007/s10900-017-0461-1 PMC6019617

[23] Viswanath K Nagler RH Bigman-Galimore CA McCauley MP Jung M Ramanadhan S The communications revolution and health inequalities in the 21st century: implications for cancer control. Cancer Epidemiol Biomarkers Prev. 2012;21(10):1701–1708. 23045545 10.1158/1055-9965.EPI-12-0852 PMC3468900

[24] Hale ED Treharne GJ Kitas GD The common-sense model of self-regulation of health and illness: how can we use it to understand and respond to our patients’ needs? Rheumatology (Oxford). 2007;46(6):904–906. 17449488 10.1093/rheumatology/kem060

[25] So J A further extension of the Extended Parallel Process Model (E-EPPM): implications of cognitive appraisal theory of emotion and dispositional coping style. Health Commun. 2013;28(1):72–83. 23330860 10.1080/10410236.2012.708633

[26] Janz NK Becker MH The health belief model: a decade later. Health Educ Q. 1984;11(1):1–47. 6392204 10.1177/109019818401100101

[27] Witte K Putting the fear back into fear appeals: the extended parallel process model. Commun Monogr. 1992;59:329–349.

[28] Viswanath K Breen N Meissner H Cancer knowledge and disparities in the information age. J Health Commun. 2006;11(suppl 1):1–17. 16641071 10.1080/10810730600637426

[29] Shrum LJ Assessing the social influence of television: a social cognition perspective on cultivation effects. Commun Res. 1995;22(4):402–429.

[30] Lee CJ The interplay between media use and interpersonal communication in the context of healthy lifestyle behaviors: reinforcing or substituting? Mass Commun Soc. 2009;13(1):48–66. 25598709 10.1080/15205430802694869 PMC4296894

[31] Dutta MJ Health information processing from television: the role of health orientation. Health Commun. 2007;21(1):1–9. 17461747 10.1080/10410230701283256

[32] Slater MD Long M Bettinghaus EP Reineke JB News coverage of cancer in the United States: a national sample of newspapers, television, and magazines. J Health Commun. 2008;13(6):523–537. 18726810 10.1080/10810730802279571 PMC3037797

[33] Rubenson D Kapp DS Getting real about NCI-designated Cancer Center advertising. Nat Rev Clin Oncol. 2017;14(4):195–196. 28266522 10.1038/nrclinonc.2017.28

[34] Gantz W Wang Z Coverage of cancer in local television news. J Cancer Educ. 2009;24(1):65–72. 19259868 10.1080/08858190802664727

[35] Wang Z Gantz W Health content in local television news. Health Commun. 2007;21(3):213–221. 17567253 10.1080/10410230701307527

[36] Pribble JM Goldstein KM Fowler EF Greenberg MJ Noel SK Howell JD Medical news for the public to use? What’s on local TV news. Am J Manag Care. 2006;12(3):170–176. 16524349

[37] Taneja H Viswanathan V Still glued to the box? Television viewing explained in a multi-platform age integrating individual and situational predictors. Int J Commun. 2014;8:2134–2159.

[38] Nielsen Group. The comparable metrics Report: QI 2016. 2016; http://www.nielsen.com/us/en/insights/reports/2016/the-comparable-metrics-report-q1-2016.html. Updated July 14, 2016. Accessed August 2018.

[39] Nielsen Group. American video habits by age, gender, and ethnicity. 2011; http://www.nielsen.com/us/en/insights/news/2011/american-video-habits-by-age-gender-and-ethnicity.html. Accessed August 2018.

[40] Raine L About 6 in 10 young adults in U.S. primarily use online streaming to watch TV. 2017; http://www.pewresearch.org/fact-tank/2017/09/13/about-6-in-10-young-adults-in-u-s-primarily-use-online-streaming-to-watch-tv/. Accessed August 2018.

[41] Niederdeppe J Hornik RC Kelly BJ Examining the dimensions of cancer-related information seeking and scanning behavior. Health Commun. 2007;22(2):153–167. 17668995 10.1080/10410230701454189

[42] Weiss BD Reed RL Kligman EW Literacy skills and communication methods of low-income older persons. Patient Educ Couns. 1995;25(2):109–119. 7659623 10.1016/0738-3991(95)00710-h

[43] Niederdeppe J Levy AG Fatalistic beliefs about cancer prevention and three prevention behaviors. Cancer Epidemiol Biomarkers Prev. 2007;16(5):998–1003. 17507628 10.1158/1055-9965.EPI-06-0608

[44] Institute TNC HINTS 4 Cycle 3. 2015; http://hints.cancer.gov/,. Accessed September 2017.

[45] Kantar Media Strategy. https://www.kantarmedia.com/us. Accessed August 2018.

[46] Hesse BW Nelson DE Kreps GL Trust and sources of health information: the impact of the Internet and its implications for health care providers: findings from the first Health Information National Trends Survey. Arch Intern Med. 2005;165(22):2618–2624. 16344419 10.1001/archinte.165.22.2618

[47] Hovick SR Understanding family health information seeking: a test of the theory of motivated information management. J Health Commun. 2014;19(1):6–23. 24117214 10.1080/10810730.2013.778369

[48] Persoskie A Ferrer RA Klein WM Association of cancer worry and perceived risk with doctor avoidance: an analysis of information avoidance in a nationally representative US sample. J Behav Med. 2014;37(5):977–987. 24072430 10.1007/s10865-013-9537-2

[49] Emanuel AS Kiviniemi MT Howell JL Avoiding cancer risk information. Soc Sci Med. 1982;147:113–120. 10.1016/j.socscimed.2015.10.058 PMC4689624 26560410

[50] Wang C O’Neill SM Rothrock N Comparison of risk perceptions and beliefs across common chronic diseases. Prev Med. 2009;48(2):197–202. 19073208 10.1016/j.ypmed.2008.11.008 PMC2720609

[51] Skinner EA Perceived Control, Motivation, & Coping. Thousand Oaks, CA: Sage; 1995.

[52] Vater LB Donohue JM Arnold R White DB Chu E Schenker Y What are cancer centers advertising to the public? A content analysis. Ann Intern Med. 2014;160(12):813–820. 24863081 10.7326/M14-0500 PMC4356527

[53] Quinn EM Corrigan MA McHugh SM Who’s talking about breast cancer? Analysis of daily breast cancer posts on the internet. Breast. 2013;22(1):24–27. 22683246 10.1016/j.breast.2012.05.001

[54] Sugawara Y Narimatsu H Hozawa A Shao L Otani K Fukao A Cancer patients on Twitter: a novel patient community on social media. BMC Res Notes. 2012;5:699. 23270426 10.1186/1756-0500-5-699 PMC3599295

[55] Xu S Markson C Costello KL Xing CY Demissie K Llanos AA Leveraging social media to promote public health knowledge: example of cancer awareness via Twitter. JMIR Public Health Surveill. 2016;2(1):e17. 27227152 10.2196/publichealth.5205 PMC4869239

[56] Himelboim I Han JY Cancer talk on twitter: community structure and information sources in breast and prostate cancer social networks. J Health Commun. 2014;19(2):210–225. 24111482 10.1080/10810730.2013.811321

[57] Armstrong N Murphy E Weaving meaning? An exploration of the interplay between lay and professional understandings of cervical cancer risk. Soc Sci Med. 2008;67(7):1074–1082. 18640758 10.1016/j.socscimed.2008.06.022

[58] Han PK Moser RP Klein WM Beckjord EB Dunlavy AC Hesse BW Predictors of perceived ambiguity about cancer prevention recommendations: sociodemographic factors and mass media exposures. Health Commun. 2009;24(8):764–772. 20183385 10.1080/10410230903242242 PMC4207435

[59] McNeish DM Modeling sparsely clustered data: design-based, model-based, and single-level methods. Psychol Methods. 2014;19(4):552–563. 25110903 10.1037/met0000024

