# Supplementary material for: Associations Between Objective Television Exposure and Cancer Perceptions in a National Sample of Adults
Source: Cancer Control. 2019 May 26;26(1):1073274819846603. doi: 10.1177/1073274819846603 (PMC6537258; doi:10.1177/1073274819846603)
Supplement: Supplemental Material, Supplemental_Table_1_and_2 - Associations Between Objective Television Exposure and Cancer Perceptions in a National Sample of Adults [file Supplemental_Table_1_and_2.pdf]

Supplemental Table 1. Weighted Univariate Analysis, Unweighted Bivariate Analysis of Perceived Worry, Comparative Risk, and Perceived Control

	Overall *		Perceived Worry			Comparative		
	N (mean)	% (SD)	β	SE	p	β	SE	
**Individual Characteristics**								
**Race/ethnicity**								
Non-Hispanic White	1584	66.88	-0.04	0.05	0.48	***0.24***	***0.04***	[illegible]
Other (ref)	1141	33.12	-	-	-	-	-	
**Household Income**								
Less than 35,000 (ref)	1098	34.69	-	-	-	-	-	
35,000 to <75,000	840	32.34	***-0.16***	***0.06***	***0.01***	***0.15***	***0.05***	[illegible]
75,000 or more	801	32.97	-0.08	0.06	0.19	***0.16***	***0.05***	[illegible]
**Education level**								
Less than high school (ref)	297	9.67	-	-	-	-	-	
High school graduate	699	24.45	***-0.18***	***0.09***	***0.05***	***0.28***	***0.08***	[illegible]
Some college	933	32.68	***-0.25***	***0.09***	***0.004***	***0.23***	***0.07***	[illegible]
Bachelor's degree	718	20.39	***-0.23***	***0.09***	***0.01***	***0.22***	***0.08***	[illegible]
Post-Bac degree	449	12.82	-0.16	0.10	0.10	***0.25***	***0.08***	[illegible]
**Gender**								
Male (ref)	1197	48.39	-	-	-	-	-	
Female	1906	51.61	***0.18***	***0.05***	***<.0001***	0.02	0.04	
**Age**	45.38	0.212	***-0.004***	***0.00***	***<0.001***	***0.01***	***0.00***	[illegible]
**Television Trust**								
Low (ref)	407	15.20	-	-	-	-	-	
Medium	2378	77.61	0.05	0.09	0.57	0.13	0.08	
High	212	7.19	-0.13	0.11	0.25	0.18	0.09	
**Designated Marketing Area**								
Annual exposure to cancer ads (hours)	443..81	0.03	0.00	0.02	0.85	0.01	0.02	
Annual dollars spent on cancer ads (mil)	83.09	0.001	-0.13	0.44	0.76	0.12	0.42	

*Overall sample is weighted and include univariate analyses, Worry, Risk, and Ability to Prevent Outcomes are not [illegible] clustering at the DMA level in bivariate analyses.
Sample size for each outcome: Overall Sample = 3,185; Perceived Worry = 2,595; Comparative Risk 2,542; Ability t[illegible]

Supplemental Table 2. Direct Effects Only Model

	Perceived Worry			Comparative Risk		
	**β**	**SE**	**p-value**	**β**	**SE**	**p-valu**[illegible]
**Intercept**	2.19	0.105	<.0001	2.46	0.13	<.000[illegible]
**Designated Marketing Area**						
Exposure to cancer ads (hours)	0.01	0.04	0.70	0.04	0.02	0.09[illegible]
Dollars spent on cancer ads (million)	-0.36	0.946	0.70	**-0.78**	**0.26**	**0.00**[illegible]
**Individual Characteristics**						
**Race/ethnicity**						
Non-Hispanic White	0.10	0.06	0.095	**0.25**	**0.05**	**<0.00**[illegible]
Other (ref)						
**Gender**						
Male (ref)						
Female	***-0.15***	***0.04***	***0.0004***	0.05	0.04	0.14[illegible]
**Age**						
**Television Trust**						
Low (ref)						
Medium	0.14	0.09	0.28	0.04	0.09	0.61[illegible]
High	0.18	0.09	0.05	-0.02	0.10	0.81[illegible]
**Estimated Parameters**						
AIC		5845.82			5325.35	
BIC		5900.10			5325.60	
-2 Log Likelihood		5811.82			5295.35	

*Models adjusted for household income and education level
